# Management of appendiceal pseudomyxoma peritonei diagnosed during pregnancy

**DOI:** 10.1186/1477-7819-7-48

**Published:** 2009-05-19

**Authors:** Erika Haase, Dal Yoo, Paul H Sugarbaker

**Affiliations:** 1 Department of Surgical Oncology, Princess Margaret Hospital, University of Toronto, Toronto, Ontario, Canada; 2 Program in Peritoneal Surface Malignancy, Washington Cancer Institute, Washington Hospital Center, Washington, District of Columbia, USA

## Abstract

**Background:**

The incidence of cancer during pregnancy is approximately 1 in 1000. The most common types encountered during pregnancy are cervical, breast and ovarian. Epithelial tumors of the appendix on the other hand are rare and account for only approximately 1% of all colorectal neoplasms; the occurrence of this neoplasm during pregnancy is extremely rare.

**Case Presentation:**

The medical history of a 30 year old woman diagnosed at 17 weeks gestation with an appendiceal mucinous tumor with large volume pseudomyxoma peritonei was presented. Her pregnancy was preserved and she had an early vaginal delivery of a healthy baby at 35 weeks. At 2 1/2 weeks postpartum the patient underwent extensive cytoreductive surgery and intraperitoneal chemotherapy. She remains disease-free 5 years after her initial diagnosis. A literature review of this clinical situation and a discussion of treatment plans were presented.

**Conclusion:**

The management of an appendiceal tumor with pseudomyxoma peritonei diagnosed during pregnancy requires full knowledge of the natural history of this disease to achieve a balance of concern for maternal survival and fetal health.

## Background

Epithelial tumors of the appendix are rare, accounting for approximately 1% of all colorectal neoplasms [1]. The tumor can range in presentation from a malignant mucocele found at routine appendectomy, to a ruptured high grade appendiceal malignancy with large volume pseudomyxoma peritonei [2]. During pregnancy, cancer occurs in approximately 1 in 1000 women, with the most common types being cervical, breast and ovarian [3]. There are only a few reports of appendiceal tumors occurring during pregnancy. Management of malignancy during pregnancy is challenging, requiring a balance of concern for maternal survival and fetal health and well-being. The management plan, which may require induced abortion, is determined by the stage of pregnancy and the predicted behavior of the cancer. We present here the medical history of a patient having an appendiceal mucinous tumor with large volume pseudomyxoma peritonei syndrome during pregnancy and the treatments she had at our institution. A review of the literature regarding this clinical situation and a discussion of treatment options are presented.

## Case presentation

A 30 year old primagravid woman at 17 weeks gestation was found on routine prenatal ultrasound to have a complex right ovarian mass. She underwent surgery and was found to have a ruptured appendiceal mucinous neoplasm with a large volume pseudomyxoma peritonei syndrome. The right ovary and appendix were removed and an omental biopsy was performed. The final pathology confirmed a well-differentiated mucinous adenocarcinoma of appendiceal origin. She recovered without incident from this surgery and was referred for assessment to the Washington Hospital Center at 26 weeks gestation. In consultation with the patient and her obstetrician it was decided to preserve the pregnancy and schedule an early vaginal delivery at 35 weeks gestation.

Following an uncomplicated vaginal delivery of a healthy baby, she underwent a staging CT. It showed no evidence of metastases within the liver parenchyma or outside of the peritoneal cavity. A large volume of mucinous cancer was imaged beneath right and left hemidiaphragm and in the pelvis. Small bowel except for the terminal ileum was spared. Preoperative tumor markers revealed an elevated CEA at 68.2 ng/mL (normal 0–5 ng/mL), CA-125 of 177 units/mL (normal 0–35 units/mL) and CA 19-9 of 361 units/mL (normal 0–37 units/mL).

At 2 1/2 weeks after delivery, the patient underwent an abdominal exploration followed by cytoreductive surgery. She had thick, densely packed tumor covering most of the parietal peritoneal surface, with an especially large volume of disease in the lesser omentum, omental bursa and surrounding the porta hepatis. The stomach and the small bowel except for the terminal ileum were spared. An extensive cytoreduction surgery was performed including total anterior parietal peritonectomy and resection of tumor in the abdominal wall scar, greater and lesser omentectomy with stripping of the omental bursa, right and left upper quadrant peritonectomies including total diaphragm stripping bilaterally, splenectomy, electroevaporation of tumor on liver capsule, cholecystectomy, and a right hemicolectomy including the distal 15 cm of terminal ileum. A total pelvic peritonectomy with abdominal hysterectomy, left salpingo-oophorectomy and rectosigmoid colon resection was performed. The peritoneal cancer index score was 28 (out of a maximum of 39), and the cytoreduction was scored as complete (residual tumor less than 2.5 mm) [4]. Intraoperative intraperitoneal heated chemotherapy was administered through the open coliseum technique, with 15 mg/m^2 ^mitomycin C at 41.5°C for 90 minutes. A Tenckhoff catheter and Jackson-Pratt drains were inserted for early postoperative intraperitoneal 5-fluorouracil chemotherapy [5]. Following completion of the hyperthermic intraoperative intraperitoneal chemotherapy an ileocolic and colorectal anastomosis was performed. The total operating time was 9 hours, and the blood loss was estimated at 350 mL, with 2 units of packed red blood cells and 4 units of fresh frozen plasma administered during the operation.

On postoperative day 1 through 5, 900 mg intraperitoneal 5-fluorouracil in 1.5% dextrose peritoneal dialysis solution was administered daily for 23 hours. The patient developed uncomplicated neutropenia on postoperative day 16, with neutrophil count of 0.9 × 10^3^/uL and a total leukocyte count of 1.3 × 10^3^/uL, which was treated successfully with granulocyte colony stimulating factor. On postoperative day 18 she was diagnosed with a left lower extremity deep venous thrombosis and was treated with intravenous heparin which was converted to warfarin prior to discharge from the hospital. She had a postoperative ileus requiring nasogastric drainage for 2 weeks and received total parenteral nutrition during this time. She was discharged in good condition on postoperative day 24.

Three weeks following surgery her tumor marker levels decreased with CEA at 0.5 ng/mL, CA 125 at 92.1 units/mL, and CA 19-9 at 10.6 units/mL. CA 125 normalized by 2 months post-operatively.

After recovery from surgery, she was treated with the Xelox regimen (Xeloda 1000 mg/m^2 ^bid for 14 days then 7 day rest and oxaliplatin 130 mg/m^2 ^intravenous over 90 minutes on day 1) for 8 cycles over 24 weeks.

In follow-up at five years after, the patient and her child are in good condition. She has had two episodes of transient small bowel obstruction treated conservatively. On her most recent clinical, radiologic and biochemical assessment at 5 years after her initial diagnosis she remains disease-free.

## Discussion

Pseudomyxoma peritonei syndrome is a rare disease registered as number 843 by the National Organization for Rare Disorders [6]. To our knowledge there is only one previous report of a disseminated appendiceal tumor occurring during pregnancy [7]. Our case of pseudomyxoma peritonei presenting in early pregnancy highlights some interesting challenges in the management of cancer in pregnancy. In this case, as in most other cancers occurring during pregnancy, there is a paucity of evidence to guide the clinician in optimal management. Maternal health and timely treatment of the malignancy is balanced by health and safety of the fetus, as many treatment interventions, including abdominal surgery, radiation, and chemotherapy, are known to be harmful to the fetus. Therefore during pregnancy, in addition to the stage and prognosis of the cancer, the stage and value of the pregnancy must also be considered.

For cancer diagnosed during the latter part of pregnancy, an optimal decision often involves a negligible delay in definitive treatment following an early delivery at about 34 weeks gestation when the risk to the premature infant is quite low. During the first trimester the fetus is most susceptible to teratogenic effects; x-rays and most chemotherapeutic agents are contraindicated. Also, spontaneous abortion is common. A long delay in treatment of an aggressive cancer is often unacceptable to the woman and her treating physicians, and a recommendation for termination of the pregnancy must be considered. During the second trimester, as in our patient, abdominal surgical procedures have the lowest risk of spontaneous abortion or premature labor. Additionally, many chemotherapeutic agents have been successfully used in the second and third trimester. Plain radiographs and even abdominal CT scans pose minimal risk to the fetus at this stage of the pregnancy. With increasing experience with abdominal MRI, this is becoming the recommended imaging modality for pregnant women with appendiceal or other gastrointestinal malignancy.

In our patient the appropriate diagnostic surgical evaluation, an appendectomy, was made at the safest time, during the second trimester. Unexpectedly at laparotomy, a diagnosis of large volume pseudomyxoma peritonei from a ruptured appendiceal mucinous carcinoma was made. The natural history of pseudomyxoma peritonei was then considered to guide our management [8]. As described by Ronnett and coworkers pseudomyxoma peritonei describes mucinous intraabdominal tumors usually of appendiceal origin with a broad spectrum of aggressiveness. The low grade appendiceal mucinous tumors (disseminated peritoneal adenomucinosis) usually have a slowly progressive course over several years. The clinical entity with the non-invasive disease is referred to as pseudomyxoma peritonei syndrome. In contrast mucinous carcinomatosis from poorly differentiated cancers of the appendix usually with signet ring morphology have an aggressive behavior, progress rapidly, and carry a worse prognosis. Well-differentiated appendiceal mucinous adenocarcinoma with pseudomyxoma peritonei, as in our patient, is a less aggressive disease, shows a less rapid progression and an intermediate prognosis.

With all appendiceal mucinous neoplasms the prognosis is dependent not only on the histologic grade of the tumor but also the completeness of cytoreduction score [9]. The volume of intraabdominal tumor as assessed by the peritoneal cancer index has no impact on prognosis [9,10]. In this patient a delay in definitive treatment undoubtedly allowed an increase in tumor volume to occur. However, since the cytoreduction was complete, minimal compromise in the prognosis was expected.

In a review of the literature, there have been 7 previous reports of appendiceal mucinous tumors occurring during pregnancy in the absence of pseudomyxoma peritonei syndrome [7,11-16]. Six were appendiceal mucinous tumors confined to the appendix and one non-mucinous appendiceal adenocarcinoma with peritoneal carcinomatosis (Table 1). In one case, the diagnosis was made in the third trimester and an early delivery was carried out prior to definitive treatment. In one case the diagnosis was made at the time of Cesarean section at term. Four patients presented with an acute abdomen and the diagnosis of appendicitis, with the correct diagnosis being revealed at laparotomy. One of these patients elected to have a therapeutic abortion prior to reoperation for a right hemicolectomy, one patient had a right hemicolectomy at initial operation at 26 weeks gestational age and went on to deliver at term, and the remaining two were treated with appendectomy. The final patient was diagnosed in very early pregnancy at the time of a spontaneous abortion, and underwent surgical treatment 3 months later after progression of disease was revealed on imaging.

**Table 1:** Case reports of appendiceal epithelial (mucinous) tumors during pregnancy.

Reference and year	Diagnosis	Gestational age at presentation	Clinical presentation	Initial treatment of appendiceal tumor	Definitive treatment of appendiceal tumor	Management and outcome of pregnancy	Patient outcome and follow-up
Haase (current case)	Well-differentiated mucinous adenocarcinoma with pseudomyxoma peritonei syndrome	17 weeks	Incidental finding on routine prenatal ultrasound	Laparotomy, right salpingo-oophorectomy, appendectomy, omental biopsy	Complete cytoreductive surgery with HIPEC and EPIC after delivery, and adjuvant chemotherapy	Early induction and vaginal delivery of healthy baby at 35 weeks	5 years, no recurrence

Sebire 2000 [7]	Moderately-differentiated appendiceal adenocarcinoma with peritoneal carcinomatosis and liver metastases	29 weeks	Lower abdominal pain and vomiting	Diagnostic workup of metastatic disease (liver biopsy)	Palliative debulking (omentectomy, appendectomy, left oophorectomy) at time of Cesarean section. Adjuvant 5-FU, epirubicin and carboplatin	Cesarean section at 30 weeks, healthy baby	6 months post treatment clinically well but residual tumor in right iliac fossa and liver

Gallo 2001 [11]	Well-differentiated mucinous cystadenocarcinoma	38 weeks	Incidental finding at Cesarean section	Appendectomy at time of Cesarean section	Right hemicolectomy after radiographic metastatic workup	Cesarean section at 38 weeks	5 years, no recurrence

Donnenfeld 1986 [12]	Perforated mucinous appendiceal adenocarcinoma	31 weeks	Acute abdomen,	Appendectomy	Right hemicolectomy 9 days postpartum	Early induction and vaginal delivery of healthy baby at 33 weeks	30 day follow up no complications

Morgan 2004 [13]	Well-differentiated mucinous adenocarcinoma, negative peritoneal washings	26 weeks	Acute abdomen	Right hemicolectomy	No further treatment	Vaginal delivery of healthy baby at term	36 months, no recurrence

Zeteroglu 2003 [14]	Mucinous appendiceal cystadenocarcinoma	21 weeks	Acute abdomen	Appendectomy	Right hemicolectomy, omentectomy	Terminated at 21 weeks	1 year, no recurrence

Casey 2003 [15]	Perforated mucinous cystadenoma	21 weeks	Acute abdomen	Appendectomy	No further treatment	miscarriage	Discharged well 4 days after surgery

Kalu 2005 [16]	Mucinous adenoma with mucocele	5 weeks	Incidental ultrasound finding at time of vaginal bleeding	Observation with serial imaging (follow-up ultrasound 3 months)	Appendectomy 3 months later when mass doubled in size	Spontaneous abortion at 6 weeks	Discharged well 4 days after surgery

HIPEC: heated intraperitoneal chemotherapy

EPIC: early postoperative intraperitoneal chemotherapy

5-FU: 5-fluorouracil

Based on our case and the previous case reports, it appears reasonable to carry out a diagnostic surgical evaluation of a mucinous appendiceal tumor during pregnancy, ideally in the second or third trimester. If a patient presents with an acute abdomen from a ruptured mucinous tumor, initial surgery should consist of a complete appendectomy and mesoappendectomy, and biopsy of omental or peritoneal tumor deposits. Since the rate of lymph node positivity in appendiceal mucinous neoplasms is less than 5%, more extensive surgery involving a right hemicolectomy at the time of initial diagnosis is not warranted and poses undue risk to the mother and fetus [17]. Definitive management of moderate or low grade appendiceal cancer with pseudomyxoma peritonei should be delayed until after delivery, as extensive cytoreduction and intraperitoneal chemotherapy would be impossible and contraindicated during pregnancy. It is reasonable to have the delivery at an earlier date, 34–35 weeks gestation, in order not to further delay treatment of appendiceal adenocarcinoma (Table 2). The method for childbirth is important if the delivery is to occur prior to definitive cytoreduction. A vaginal delivery is required. A Cesarean section is contraindicated. An abdominal incision for Cesarean section will allow for mucinous cancer cells to implant and progress within the abdominal incision and parametrial tissues. This would potentially compromise the completeness of cytoreduction and the likelihood of a curative result [18]. If a Caesarian section is necessary for obstetrical reasons, a midline incision should be used, which can be excised with subsequent cytoreductive surgery. Stage for stage, one would postulate that pregnant cancer patients with pseudomyxoma peritonei may have similar outcomes as non-pregnant patients. With optimal management including complete cytoreduction and intraperitoneal chemotherapy, patients with pseudomyxoma peritonei from well-differentiated appendiceal mucinous carcinoma will have a 15-year survival of 50% [9].

**Table 2:** Suggested management strategy for pseudomyxoma peritonei syndrome occurring during pregnancy.

	**Aggressive Malignancy**		**Low-Moderate Grade Malignancy**	
	
Week of Pregnancy	Diagnostic Tests	CRS + HIPEC	Diagnostic Tests	CRS + HIPEC
0–12	Unsafe	Consider pregnancy termination	Unsafe	Delay treatment to 35 weeks

13–27	Safe	Consider pregnancy termination	Safe	Delay treatment to 35 weeks

28–40	Safe	Delay treatment to 35 weeks	Safe	Delay treatment to term

(CRS = cytoreductive surgery, HIPEC = hyperthermic intraperitoneal chemotherapy)

Koops and colleagues wrote about pseudomyxoma peritonei syndrome diagnosed in women who were attempting pregnancy (Koops A, Smeenk RM, Zoetmulder FAN, Hoek A. Pseudomyxoma peritonei and pregnancy. Report of two cases, unpublished data). They recommend that definitive treatment of the appendiceal tumor be delayed to allow pregnancy to occur if the disease was minimally aggressive. In patients with progressive disease their experience with delay led to an advanced, untreatable disease state.

## Conclusion

In patients with slow or moderate advance of disease, the pregnancy (or pregnancy wish) should be allowed to proceed to a vaginal delivery. In patients with a rapid progression, termination of the pregnancy and definitive treatment may be necessary to protect the mother.

## Abbreviations

CEA: carcinoembryonic antigen; CA 19-9: cancer antigen 19-9; CA 125: cancer antigen 125; CT: computed tomography.

## Consent

Written informed consent was obtained from the patient for publication of this case report and any accompanying images. A copy of the written consent is available for review by the Editor-in-Chief of this journal.

## Competing interests

The authors declare that they have no competing interests.

## Authors' contributions

All Authors made substantial contributions to the concept, design, acquisition of data, analysis and interpretation of data, drafting and revising the intellectual content of the manuscript. All Authors read and approved the final manuscript.
